# Ventricular septal rupture following myocardial infarction: A potentially fatal complication

**DOI:** 10.1002/ccr3.1986

**Published:** 2019-01-09

**Authors:** Athanasios Saplaouras, Antigoni Sakellaropoulou, Panagiotis Mililis, George Bazoukis, Angelos‐Michail Kolokathis, Eleni Konstantinidou, Aikaterini Anagnostou, Niki Panagopoulou, Konstantinos Vlachos, Ioannis Lakoumentas, Antonios Sideris

**Affiliations:** ^1^ Second Department of Cardiology "Evangelismos" General Hospital of Athens Athens Greece

**Keywords:** complication, infraction, myocardial, rupture, septal, ventricular

## Abstract

Mechanical complications of acute myocardial infarction include ventricular septal rupture (VSR), free wall rupture, and ischemic mitral regurgitation. Postinfarction VSR is a rare but serious complication of myocardial infarction. VSR has a lower incidence in the era of new reperfusion therapies. However, clinicians should be aware of this potentially fatal complication as the mortality remains extremely high. Early diagnosis and treatment are the cornerstones of achieving a better outcome.

## 1 DESCRIPTION OF CASE

**Figure 1:**
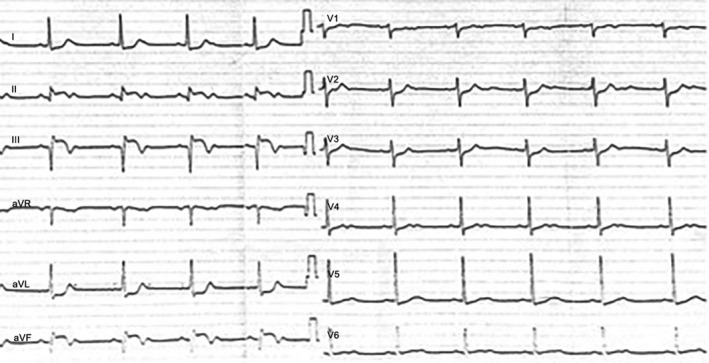
Electrocardiogram of the patient at the time of admission showed second‐degree atrioventricular block (2:1), and Q‐waves in the inferior lead with ST‐segment elevation >2 mm

**Figure 2:**
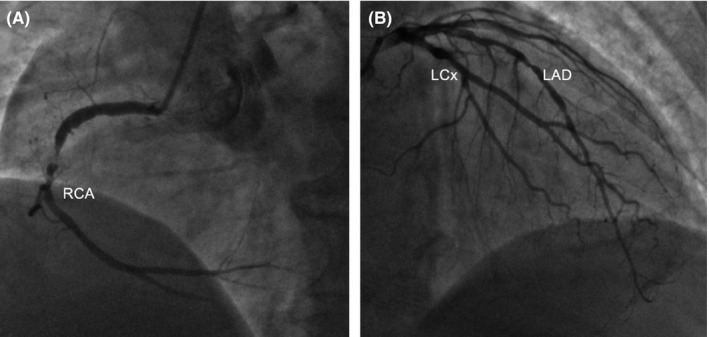
Coronary angiography (A) LAO cranial view: right coronary artery (RCA) with subtotal occlusion in the mid‐segment. (B) RAO caudal view: left anterior descending artery (LAD) with a proximal and mid 80% stenoses, left circumflex artery (LCx) with a proximal 70% stenosis and mid 80% stenosis

**Figure 3:**
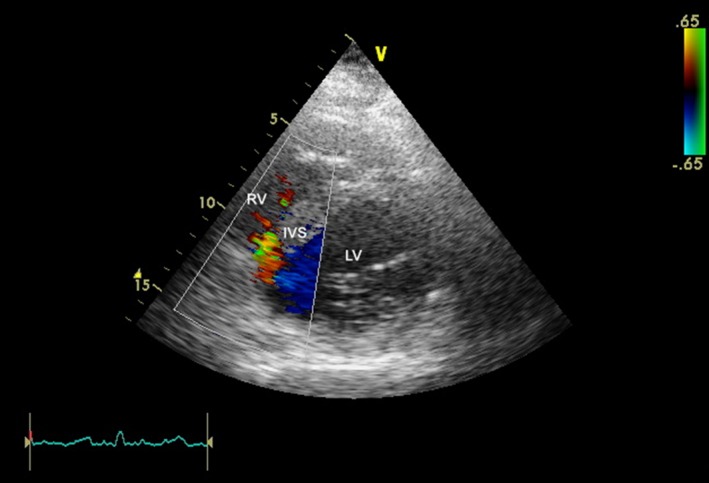
Presence of ventricular septal defect as seen in 2‐dimensional transthoracic echocardiography in parasternal short‐axis view (PTSAX)

A 73‐year‐old man with medical history of hypertension and diabetes mellitus presented to the emergency department of our hospital complaining of fatigue and dizziness. He reported having an episode of epigastric pain of two‐hour duration a week ago. His vital signs on admission were as follows: blood pressure 110/70 mm Hg, heart rate 52 beats per minute, and SpO_2 _98%. Cardiac and pulmonary auscultation were unremarkable. Electrocardiogram revealed sinus rhythm with intermittent second‐degree atrioventricular block (2:1), and Q‐waves in the inferior lead with ST‐segment elevation >2 mm (Figure 1). On admission, levels of hs‐Troponin T were elevated (2657 μg/L) as well as the rest of myocardial injury markers. Transthoracic echocardiogram (TTE) showed reduced left ventricular ejection fraction, estimated about 35%‐40%, with inferior wall akinesis. The patient underwent coronary angiography the next day, which revealed triple vessel disease. The left anterior descending artery had two 80% stenoses in the proximal and mid‐segments. The left circumflex artery had a 70% proximal stenosis and an 80% stenosis in the mid portion. In addition, the right coronary artery had subtotal occlusion at the mid‐segment (Figure 2). The heart team recommended coronary artery bypass grafting surgery, so the patient was admitted to the intensive care unit with the diagnosis of subacute inferior myocardial infarction. He remained asymptomatic and hemodynamically stable during the first two days of hospitalization, with myocardial damage enzymes following a downward trend. On the third day, he developed acute dyspnea accompanied by hemodynamic instability. The patient was initiated on inotropic agents. Cardiac auscultation revealed a holosystolic murmur that was absent the days before and that was most clearly heard in the left lower sternal border. TTE did not reveal acute mitral regurgitation or left‐to‐right shunt despite the high clinical suspicion of a mechanical complication. The patient immediately underwent right‐side catheterization which supported the diagnosis of VSR (right atrial: pH: 7.20, pCO_2_: 31 mm Hg, pO_2_: 33 mm Hg, SPO_2_: 37.5%, ${\text{HCO}}_{\text{3}}^{\;\text{-}\;}$:12 and right ventricle: pH: 7.20, pCO_2_: 20 mm Hg, pO_2_: 63.5 mm Hg, SPO2: 84%, ${\text{HCO}}_{\text{3}}^{\;\text{-}\;}$:12). A repeat TTE revealed the communication between right and left ventricle with color Doppler, possibly due to the progression of the septal rupture (Figure 3). Despite high doses of inotropic agents and optimal medical therapy, the patient remained hemodynamically unstable. An emergent surgical repair was decided by the cardiothoracic team. During the preparation of intra‐aortic balloon pump insertion, the patient suffered a cardiac arrest. After the second cycle of cardiopulmonary, resuscitation return of spontaneous circulation was detected and the intra‐aortic balloon pump was positioned. The patient suffered a second cardiac arrest and died despite the resuscitation attempt, before undergoing surgery.

## 2 DISCUSSION

The incidence of VSR has declined to 0.17%‐0.31% after the initiation of new reperfusion therapies. Among patients with cardiogenic shock, it has an incidence of 3.9%. The current mortality of this mechanical complication remains extremely high (41%‐80%) and unchanged during the last decades.^1^, ^2^ The factors that have been associated with VSR are advanced age, anterior myocardial infarction, female gender, and lack of smoking history.^3^ Our patient presented with subacute inferior myocardial infarction and the VSR developed on day 3 of his hospitalization. The median time from the onset of myocardial infarction to septal rupture was 1 day in the GUSTO‐I trial (range 0‐47; 94% of cases were diagnosed within 1 week)^3^ and 16 hours in the SHOCK trial.^4^

Hemodynamically, the presence of VSR gives rise to a left‐to‐right shunt, followed by right ventricular volume overload, increased pulmonary blood flow, and secondary volume overload of the left atrium and ventricle which in turns results to left ventricular deterioration.^2^ Cross‐sectional echocardiography with a sensitivity of about 40% and Doppler ultrasound with sensitivity close to 100% are rapid and well‐established techniques for diagnosing VSR after myocardial infarction.^5^, ^6^, ^7^ The angiographic findings in our patient are similar to those of GUSTO‐I trial. Specifically, in the GUSTO‐I study, total occlusion of the infarct‐related artery was documented in 57% of patients with VSR, as compared with 18% of those without VSR.^3^ The same study showed that the left anterior descending artery was the most associated artery with VSR. Furthermore, some studies have shown a higher incidence of VSR in patients with multivessel disease, while other studies showed a high prevalence of single vessel disease among patients with VSR.^2^ In our patient, the coronary angiography showed a subtotal occlusion of the infarct‐related artery which was the right coronary artery.

Two types of VSR can be recognized according to their pathological characteristics. Simple ruptures result by direct through‐and‐through defects and usually complicate anterior myocardial infarctions. Complex ruptures are associated with serpiginous dissection tracts remote of the primary myocardial infarction site and are more common after inferior myocardial infarctions.^8^, ^9^

The management of VSR can be separated into medical therapy and mechanical closure. The medical therapy aims to the temporary stabilization of the patient and consists of oxygenation, diuretics, inotropic agents, and afterload reduction supported by the use of intra‐aortic balloon pump. Most patients require surgical management with mechanical closure of the rupture. Different techniques as well the factors that can influence the surgical outcomes have been studied and further research is ongoing.^10^, ^11^, ^12^ Newer techniques which avoid direct incision of the ventricles can be used in selected patients.^13^, ^14^ Coronary artery bypass grafting should be performed in patients with multivessel coronary artery disease.^3^ Another interesting finding from GUSTO‐I trial was that patients with VSR selected for surgical repair have better outcomes than patients treated medically (30‐day mortality, 47% vs 94%).^3^ Concerning the time of surgery, improved outcomes were seen with delayed surgery: 18.4% mortality for patients who underwent surgery after 7 days vs 54.1% mortality for those who underwent surgery within 7 days.^1^ The improved outcome with delayed intervention could be related to evolution of the infarct, which allows a more effective surgical repair or consists a survival bias, as an early surgery is usually performed on individuals with hemodynamic instability.^15^ An early hemodynamic stabilization with Impella implantation has been proposed in order to delay surgery and reduce the surgical risk.^15^

## CONFLICT OF INTEREST

The authors declare no conflict of interest.

## AUTHOR CONTRIBUTION

AS: involved in management of the patient, wrote the draft, and final approval. AS and PM: involved in management of the patient, major revision, final approval. GB, A‐MK, EK, AA, AA, NP, KV, IL, and AS: involved in management of the patient and final approval.
